# A177 AUTOIMMUNE ENTEROPATHY – AN UNCOMMON CAUSE OF MALNUTRITION

**DOI:** 10.1093/jcag/gwad061.177

**Published:** 2024-02-14

**Authors:** G S Minhas, A Bukhari, F Tse

**Affiliations:** Gastroenterology, McMaster University, Hamilton, ON, Canada; Gastroenterology, McMaster University, Hamilton, ON, Canada; Gastroenterology, McMaster University, Hamilton, ON, Canada

## Abstract

**Background:**

Autoimmune Enteropathy is a rare condition characterized by clinical symptoms of intractable diarrhea, vomiting, and weight loss that often results in malnutrition. It is associated with other autoimmune conditions from primary sclerosing cholangitis to type 1 diabetes and autoimmune thyroid disease. The epidemiology of AIE in adults is not well described. It is detailed more in the pediatric population, particularly early in life, the incidence is estimated at less than 1 in 100,000 infants.

**Aims:**

Aims: To review a case of malnutrition secondary to Autoimmune enteropathy seen at our centre, and review the limited literature for treatment.

**Methods:**

We did a case report presentation on an inpatient seen at our Tertiary care center in Hamilton, Ontario

We reviewed the limited literature regarding treatment of autoimmune enteropathy in adults and modelled our treatment based on this.

**Results:**

We describe the case of a 63-year-old male in Ontario, Canada whereby he was initially diagnosed with ulcerative colitis, but did not respond to therapy – with the final diagnosis being autoimmune enteropathy. He was first consulted to Gastroenterology with colonoscopy consistent with mayo III disease, and biopsies showing chronic active colitis. He did not improve despite 5-ASA compounds, oral steroids, and trials of IV steroids. He underwent multiple admissions for chronic watery diarrhea, and subsequent treatment with IV steroids. He was started on an Infliximab biosimilar with little to no clinical response, and subsequently referral to our centre for evaluation of worsening malnutrition. Our patient’s symptoms mirrored much of what was initially described in the literature in 1985 with significant unremitting diarrhea, and dependence on TPN due to malabsorption. He underwent an EGD which demonstrated villous blunting, and apoptotic bodies – reviewed by a GI-specialized pathologist who found it was consistent with autoimmune enteropathy. His serology testing revealed a weakly positive TTG, HLA testing was completed showed a HLA DQ2 positivity and a gluten free diet was reccomended. His symptoms improved drastically from 10-12 bowels movements daily, to 1-2 daily within two weeks of treatment.

The limited literature surrounding treatment of autoimmune enteropathy was reviewed and the patient was started on oral budesonide in a tapering dose with significant clinical improvement as described in a retrospective case-control study in 2017.

**Conclusions:**

Autoimmune enteropathy is a rare cause of malnutrition in adult patients. It is often associated with other autoimmune conditions, withsmall bowel histology showing villous atrophy, villous blunting, apoptotic bodies, and a relatively low amount of lymphocytic infiltration. The literature on treatment is limited, our patient responded to initial treatment of gluten removal for underlying celiac disease combined with oral budesonide.

**Funding Agencies:**

None

